# What determines sclerobiont colonization on marine mollusk shells?

**DOI:** 10.1371/journal.pone.0184745

**Published:** 2017-09-13

**Authors:** Vanessa Ochi Agostini, Matias do Nascimento Ritter, Alexandre José Macedo, Erik Muxagata, Fernando Erthal

**Affiliations:** 1 Laboratório de Zooplâncton, Instituto de Oceanografia, Universidade Federal do Rio Grande (FURG), Rio Grande, Rio Grande do Sul, Brazil; 2 Programa de Pós-Graduação em Oceanografia Biológica, Instituto de Oceanografia, Universidade Federal do Rio Grande (FURG), Rio Grande, Rio Grande do Sul, Brazil; 3 Programa de Pós-Graduação em Geociências, Instituto de Geociências, Universidade Federal do Rio Grande do Sul, Porto Alegre, Rio Grande do Sul, Brazil; 4 Faculdade de Farmácia and Centro de Biotecnologia, Universidade Federal do Rio Grande do Sul, Porto Alegre, Rio Grande do Sul, Brazil; 5 Departamento de Paleontologia e Estratigrafia, Instituto de Geociências, Universidade Federal do Rio Grande do Sul, Porto Alegre, Rio Grande do Sul, Brazil; UPMC, FRANCE

## Abstract

Empty mollusk shells may act as colonization surfaces for sclerobionts depending on the physical, chemical, and biological attributes of the shells. However, the main factors that can affect the establishment of an organism on hard substrates and the colonization patterns on modern and time-averaged shells remain unclear. Using experimental and field approaches, we compared sclerobiont (i.e., bacteria and invertebrate) colonization patterns on the exposed shells (internal and external sides) of three bivalve species (*Anadara brasiliana*, *Mactra isabelleana*, and *Amarilladesma mactroides*) with different external shell textures. In addition, we evaluated the influence of the host characteristics (mode of life, body size, color alteration, external and internal ornamentation and mineralogy) of sclerobionts on dead mollusk shells (bivalve and gastropod) collected from the Southern Brazilian coast. Finally, we compared field observations with experiments to evaluate how the biological signs of the present-day invertebrate settlements are preserved in molluscan death assemblages (incipient fossil record) in a subtropical shallow coastal setting. The results enhance our understanding of sclerobiont colonization over modern and paleoecology perspectives. The data suggest that sclerobiont settlement is enhanced by (*i*) high(er) biofilm bacteria density, which is more attracted to surfaces with high ornamentation; (*ii*) heterogeneous internal and external shell surface; (*iii*) shallow infaunal or attached epifaunal life modes; (*iv*) colorful or post-mortem oxidized shell surfaces; (*v*) shell size (<50 mm^2^ or >1,351 mm^2^); and (*vi*) calcitic mineralogy. Although the biofilm bacteria density, shell size, and texture are considered the most important factors, the effects of other covarying attributes should also be considered. We observed a similar pattern of sclerobiont colonization frequency over modern and paleoecology perspectives, with an increase of invertebrates occurring on textured bivalve shells. This study demonstrates how bacterial biofilms may influence sclerobiont colonization on biological hosts (mollusks), and shows how ecological relationships in marine organisms may be relevant for interpreting the fossil record of sclerobionts.

## Introduction

The biological remains of invertebrates and vertebrates (shells, carapace, skeletons, and bones) may act as colonization surfaces for invertebrates, especially on continental shelves covered by unconsolidated substrates. Similarly, those remains act as colonization islands in these environments and provide a supply of invertebrate larvae, which are essential for population persistence in such regions. These biological remains are dominated by mollusk shells that can remain for long time intervals at the sediment-water interface due to their relatively high durability (or in a safe zone of the taphonomically-active, [1]). Thus, mollusk shells provide a valuable archive of current and past generations of organisms and preserve the biological signals despite the time-averaging of generations and taphonomic bias ([2] and references therein).

The intriguing relation in sclerobiont colonization (encrustation and bioerosion caused by epi- and endobiont organisms, respectively, [3]) between a host and its colonizers has been widely debated by several studies concerning the modern marine environments as well as those related to the fossil record (e.g., [4–9] and references therein). On a paleontological perspective, the encrusting communities on hard substrates changed throughout the Phanerozoic (since the Ordovician when were first expressed [9]), which provides a straightforward record of competition and interactions (e.g., [10]). As a large proportion of sclerobiont species possess highly preservable skeletons, they exhibit relatively good fossilization potential and retain the spatial structure of the encrusting communities [9]. Additionally, the ecological and taphonomical relationships of modern encrusting organisms have been the focus of numerous studies (e.g., [11–13], and references therein). Ancient biological interactions have also been explored to understand the evolutionary relationships modulated by predation [14–16], and how encrustation and bioerosion affect the interpretation of the fossil record (e.g., [8; 17–19] and references therein).

The invertebrates associated with sclerobiont colonization can be found in the zooplankton community and are mostly represented by organisms with a meroplanktonic life-cycle (i.e., barnacles, some mollusks). Meroplankton expend part of their lives in the water column as larvae drifting with ocean currents and the other part as adults in benthic or nektonic environments [20]. Holoplanktonic (i.e., some copepods) and thycoplanktonic (i.e., amphipods) invertebrates can also be recorded on hard substrates and are classified as vagile or fouling companion fauna [21–24].

There are many studies that have compared sclerobiont colonization patterns between different taxa and substrates [11]. However, there is still no consensus on the main factors that can affect invertebrate colonization on biological substrates such as shells, carapaces, and bones. However, the surface texture has frequently been cited [25–31] together with biological factors, such as competition by recourses [32, 33], conspecific presence [34, 35], and ecological inter-specific interactions [11, 36], to induce or repulse settlement. Experimental arrays conducted on non-biological hard substrates such as steel and concrete have demonstrated that invertebrate settlement might be positively [37–39] or negatively [39–41] influenced by bacterial biofilm. These biofilms are composed of multiple species of bacteria attached to a substratum covered by an extracellular polymeric matrix, and their development can change the attractiveness of a hard substrate to periphyton, protozooplankton, seaweed and invertebrates [11, 42–44].

In this study, an experimental approach was used to compare the zooplankton and bacterial biofilm colonization potentials on the shell of three species of bivalves with different external textures. Furthermore, we evaluated the encrustation and bioerosion of a marine subtropical deposit to assess the possible selectivity of sclerobionts in the fouling process on time-averaged shells (accumulation of non-contemporaneous individuals in an assemblage; see review in [2]), which simulated the upper limit of the taphonomically active zone (TAZ) [1]. The goals were to assess the main factors that affect the colonization process on shells and observe how much of the biological signal from present-day invertebrate larvae settlement is preserved in the empty molluscan shells (death assemblage–incipient fossil record) over ecological and paleoecological perspectives.

## Materials and methods

### Ethics statement

“Concheiros Beach” is located on the coast of Southern Brazilian, and is not included in the list of sites of natural interest protected by law. Endangered mollusk taxa have not been reported at the sampled location. Consequently, the field study did not involve endangered or protected species. Live molluscan specimens were not collected in this study, and special permits were not required to obtain empty shell material for scientific research in the study area. This study is supported by the “Biofouling process under subtropical coastal conditions”, project supervised by Dr. Erik Muxagata and approved by PROPESP/FURG (http://www.propesp.furg.br) (process 673520/2013, 06/2013 to 06/2017). The collect of zooplankton is permitted under the *Instituto Chico Mendes de Conservação da Biodiversidade* (*Sistema de Autorização e Informação em Biodiversidade*) permanent authorization number 1907371. The data from this study have been archived as a PLoS One online-access appendix (S1, S2 and S3 Data).

### Experiment observations: Zooplankton colonization

**Fig 1:**
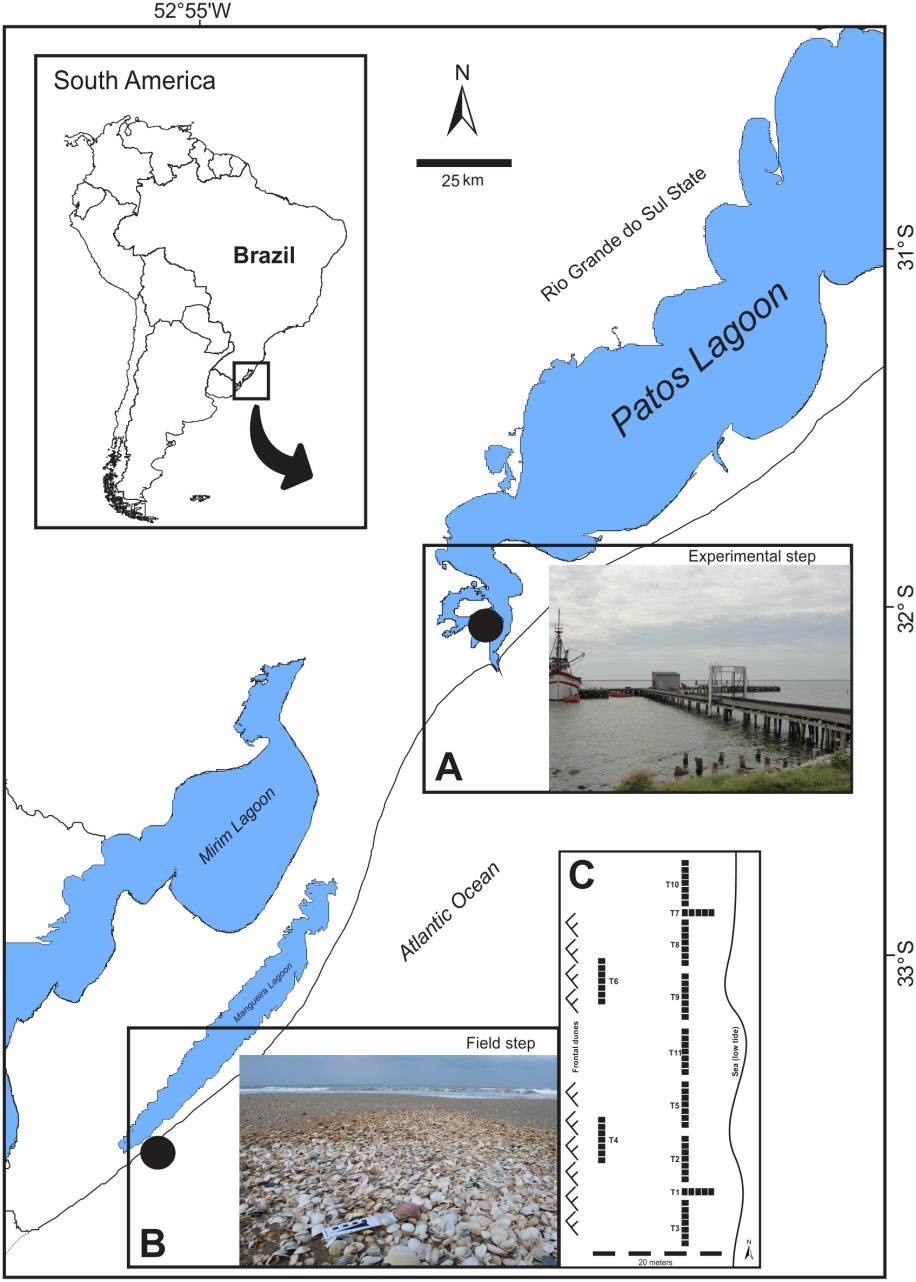
Study area on the southern Brazilian coast. (A) Patos Lagoon estuary where the experimental step was conducted. (B) “Concheiros Beach” where the samples were collected.

Shells of *Anadara brasiliana* (Lamarck, 1819), *Mactra isabelleana* d’Orbigny 1846 and *Amarilladesma mactroides* (Reeve, 1854) (S1 Fig) were chosen for this experiment since they were abundant and had distinct external textures with similar colors (white = natural or reduced color). All shells (36 specimens, 12 of each species) were gathered from Concheiros Beach, RS, Brazil (Fig 1B). The shells were immersed in sterile water in the laboratory, and three pulses of 20 kHz of a Cole-Parmer^®^ 4710 ultrasonic homogenizer were applied for 15 seconds on each side of the shell [45] to detach the biofilm. Each shell was previously observed under a dissecting microscope (Olympus BH-2) to ensure that there were no unique marks (i.e., predation, bioerosion, encrustation, fragmentation), and categorized using their external ornamentation (0 = *A*. *mactroides*; 2 = *M*. *isabelleana*; 3 = *A*. *brasiliana*) using criteria taken from the literature (references in Table 1).

**Table 1:** Categorical variables measured in this study.

Ecological variables	Key	More information/ Methodology
**Class**	0 = Gastropoda; 1 = Bivalvia	Rios [47]
**Surface size class (mm**^**2**^**)**	<50; 51–150; 151–450; 451–1350; >1350	Rodland et al. [19]
**Habitat of origin**	0 = deep infaunal; 1 = shallow infaunal; 2 = attached infaunal; 3 = free-living epifaunal	Rios [47], Mikkelsen and Bieler [48]
**Mineralogy**	1 = calcite; 2 *=* aragonite; 3 = bimineralic	Mikkelsen and Bieler [48]
**Sclerobionts (bioerosion or encrustation)**	0 = absent; 1 = present; 1.1 = drill; 1.2 = sponge; 1.3 = worn; 1.4 = bryozoan; 1.5 = ‘fungae’; 1.6 = polychaete; 1.7 = bivalve; 1.8 = barnacle; 1.9 = foraminifera; 1.10 = algae; 1.11 = hydrozoan; 1.12 = unidentified	Lecinsky et al. [49]
**Secondary color (or color alteration)**^a^	0 = color lost; 1 = natural; 2 = oxidized color; 3 = reduced color	Callender et al. [4] and Best [50]
**External ornamentation (complexity degree)**	0 = absent; 1 = low; 2 = average; 3 = high	Carl et al. [30]
**Internal ornamentation**	0 = absent; 1 = present	Carl et al. [30]

^a^oxidized colors (cream, yellow, ochre, and red); reduced colors (white, gray, and black)

Later, the shells were placed in six bowls (20 cm in diameter, 18 cm in height) filled with estuarine water (filtered through 20 μm mesh) to a height of 10 cm and kept at a constant salinity (23±2), temperature (25°C) and photoperiod (14L:10D). These conditions were chosen to simulate the current subtropical conditions found in this region. A 5 cm-thick layer of natural estuarine sediment was included as substrate at the bottom of each bowl to simulate the reintroduction of the shells to the upper part of the taphonomically active zone [1]. The shells were inserted in the sediment (~2 cm) in a way that allowed both the internal (concave) and the external (convex) sides to be exposed to the six replicates, and the shells were arranged in an interleaved manner (S2 Fig). The sizes of the shells belonging to the same species were similar, but the sizes were different among species (21 to 22 mm^2^ for *A*. *mactroides*, 7 to 8 mm^2^ for *A*. *brasiliana* and 9 to 10 mm^2^ for *M*. *isabelleana*). Thus, the zooplankton colonization density on shells was standardized to 25 mm^2^. Once a week, the seawater was partially renewed (50%), and the zooplankton community was also replaced. A supply of fresh plankton for the experimental study was collected from the channel the Patos Lagoon estuary, which is located in Rio Grande on the southern Brazilian coast (32°08’53”S– 52°06’03”W) (Fig 1A). Two samples were collected using a conventional conical plankton net (200 μm of mesh) equipped with a flowmeter. After collection, the plankton samples were filtered through a 500 μm mesh net to remove the large planktonic predators. One sample was split into six equal parts (Motoda splitter) and placed into the bowls, while the other sample was fixed (formaldehyde 4%) to analyze the potential of the zooplankton to colonize the shells.

To assess the zooplankton potential (the relationship between the invertebrates present in the water column and the colonizers on available substrates), the composition in each zooplankton sample was estimated from aliquots (1–5% of the sample) counted on a Bogorov chamber, and the results were compared to the occurrence on the shells. A General Linear Model (GLM) analysis was performed to evaluate the differences between the density of the settled zooplankton and the richness of the bivalve shell species and the exposed shell side (internal and external). A post hoc Tukey test followed the analyses. A simple regression was applied to evaluate the correlation between the settled zooplankton densities on the different shells textures.

### Experiment observations: Microbial biofilm colonization

To evaluate shell colonization by bacterial biofilms, five shells of each bivalve species (*A*. *brasiliana*, *M*. *isabelleana* and *A*. *mactroides*) were sterilized (see the section Experiment Observations: zooplankton colonization section) and attached to a pier located in the channel of the Patos Lagoon estuary (Fig 1B) during the austral summer of 2014 (salinity 23±2, temperature 25°C and photoperiod 14L:10D) (S2 Fig). The sizes of the shells were the same as those used in the laboratory experiment. The shells were recovered after five weeks of exposure and immersed in a sterile formaldehyde 4% solution (50 Ml) to fix the biofilm. In the laboratory, the biofilm was detached using three pulses of 20 kHz for 15 seconds on each side of the shells with a Cole-Parmer^®^ 4710 –ultrasonic homogenizer [45].

The biofilm bacteria density (bact cm^-2^) was estimated using a flow cytometer (BD FACSVerse^™^). The comparative sizes (μm) and complexities of the cells were measured using a Forward Light Scatter (FSC-A) and a Light Side Scatter (SSC-A), using spherical beads as the pattern [51–53]. However, the precise value of bacteria cell size was also estimated using epifluorescence microscopy, which is considered a more accurate technique than flow cytometry [54]. A total of 100 bacterial cells were measured for each bivalve species. The bacterial biomass (pg C cell^-1^) was calculated using the allometric biovolume (μm^3^) conversion factors proposed by Norland [55] and Sun and Liu [56].

To evaluate the microbial community, the biological material in suspension obtained from each shell was filtered (1 mL) through polycarbonate filters (darkened with Irgalan Black), stained with acridine orange (1%) and viewed under an epifluorescence microscope (Zeiss Axioplan) at 1000X magnification. The bacterial morphotypes were classified according to Zaritski [57]. The observations of the presence or absence of fungi and periphyton followed the same methodology.

The GLM analysis was performed to evaluate the biofilm bacterial density on the different bivalve shells. The model was adapted to the Poisson distribution with a “log” link function. Post hoc Tukey tests followed the analyses. Simple and multiple regressions were applied to evaluate the correlation between the settled zooplankton density and the biofilm bacteria density on the different shell textures.

### Field observations: Mollusk assemblages

To quantify the biofouling on the time-averaged mollusk assemblages, samples were collected from Concheiros Beach (Fig 1B; 33°32’6” S– 53°5’37” W) on the Southern Brazilian coast in December 2013. This locality is well known to have dense bioclastic concentrations formed by shells mobilized from the inner continental shelf during storm events. Five to seven replicate quadrats (300 x 300 cm) were delimited, and the uppermost 5-cm sediment layer was collected. A total of 11 transects were sampled. Two transects were placed at a distance of 20 meters from the lowest sea level height in the upper supralittoral zone parallel to the shoreline; two were placed in the intertidal area perpendicular to the coastline, and the remaining seven transects were placed in the lower supralittoral zone parallel to the shore (Fig 1C).

All shell remains collected from each quadrat were identified and stored in plastic bags and taken to the laboratory, where they were washed in fresh water and sieved using 500 μm meshes. Host and fouling organisms were identified to the lowest possible taxonomic level according to Roland et al. [5], Brett et al. [8, 7], Rios [47], Buckup and Bond-Buckup [58], Lopes [59], Barclay et al. [60]. Host organisms were characterized according to their (*i*) life modes (deep infauna, shallow infauna, free-living epifauna, or attached epifauna), (*ii*) ornamentation complexity, both internal (present or absent) and external, with complexity varying from absent, little, average to high, (*iii*) predominant mineralogy (aragonite, calcite, bimineralic) and (*iv*) categorical color (natural, reduced, oxidized) (Table 1). The marks left by fouling organisms were also considered (bioerosion); they were identified and quantified under a stereoscopic microscope to determine presence or absence, coverage percentage, and the location of the colonization on the shell (internal or external). Taphonomic analyses were also carried out on all shells (S3 and S4 Data, S1 Table).

The area-size and shell data were transformed into categorical variables used to observe the occurrence frequency (%) of sclerobionts (bioerosion + encrustation) between different life modes, shell sizes, colors, ornamentations, and mineralogy. The GLM analysis was carried out to test for significant differences. The model was adapted to the data using a binomial/multinomial distribution with a “logit” link function. Post hoc Tukey tests followed the analyses. A Spearman rank correlation was performed to verify the relationship between the different categorical variables and identify any possible covariances among them. All analyses were carried out in R [61].

## Results

### Experiment observation: Zooplankton colonization

**Fig 2:**
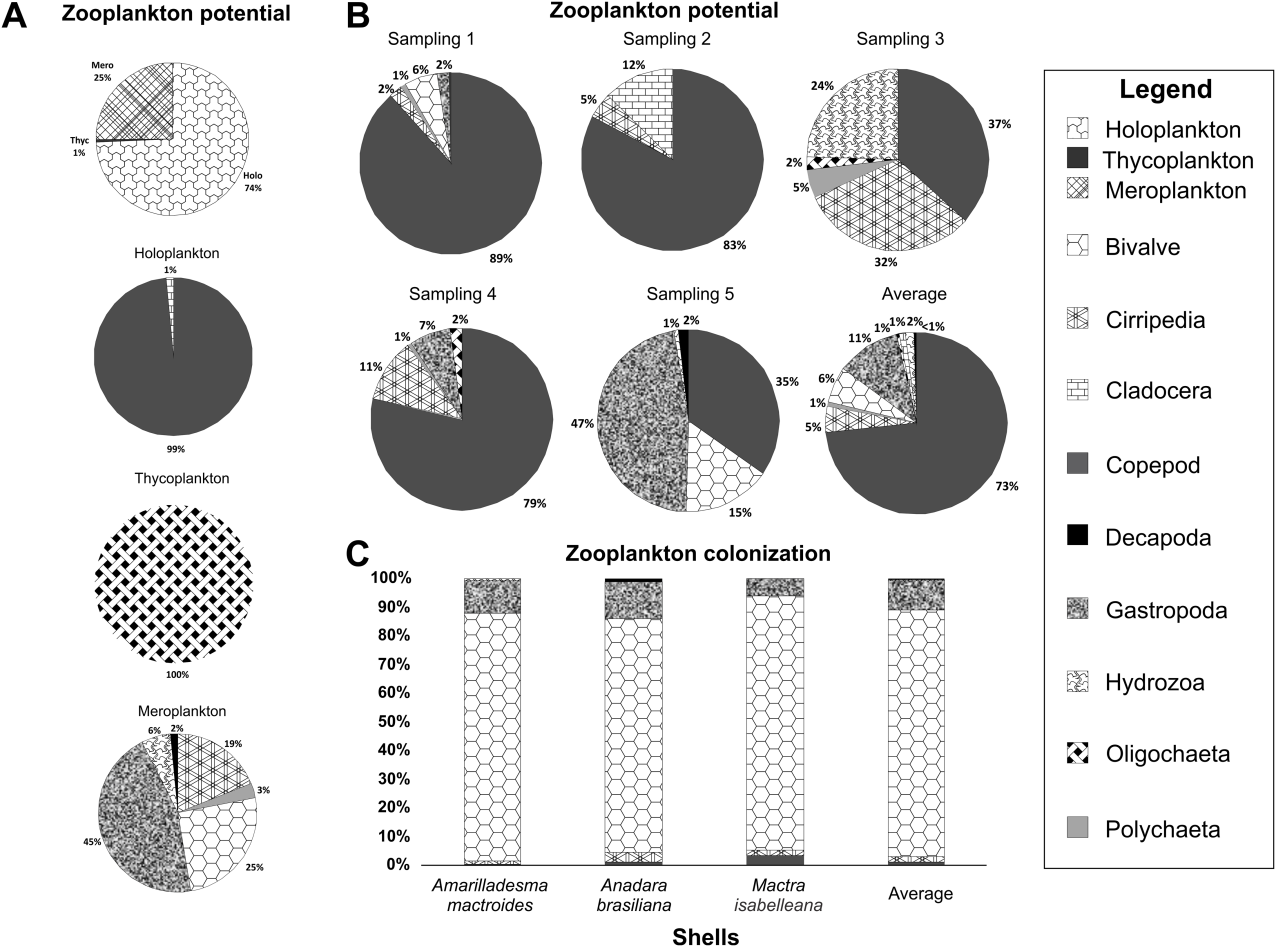
Zooplankton potential colonization on shells. (A) Total occurrence frequency (%) of holoplankton, thycoplankton, and meroplankton in zooplankton samples. (B) Zooplankton potential on sampled colonizing shells. (C) Settled zooplankton (%) on shells.

**Fig 3:**
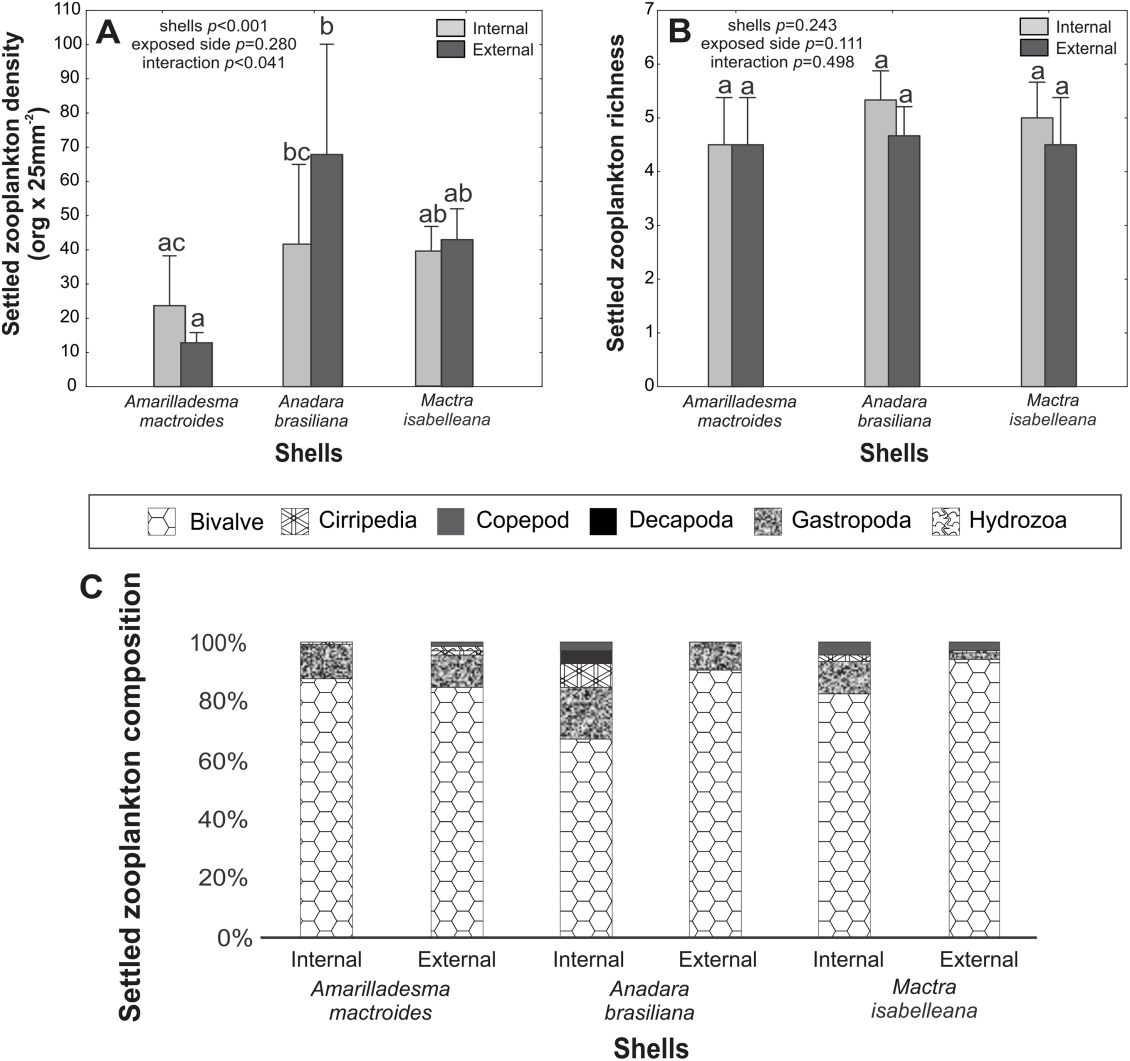
Zooplankton colonization on shells. (A) The colonization density on the internal and external surfaces of different shells. (B) The richness of colonizers on internal and external surfaces. (C) Settled zooplankton composition (%) on different shells sides. The vertical lines denote the 95% confidence intervals (standard error*1.96), and the lowercase letters indicate similarities (the same letters) or significant differences (different letters) between the shells (Tukey test).

The meroplanktonic components represented 25% (3,434 organisms m^-3^) of the zooplankton samples collected from the channel in the Patos Lagoon estuary. Holoplankton components represented 74% of the samples and thycoplankton represented 1%. However, the meroplankton contained a higher number of groups than the other components (Fig 2A). The dominant meroplanktonic organisms were gastropods (339±426 org m^-3^), followed by bivalves (190±228 org m^-3^), barnacles (139±87 org m^-3^), hydromedusae (29±36 org m^-3^), polychaetes (22±22 org m^-3^) and decapods (10±17 org m^-3^). During the experiment, the natural zooplankton community changed their composition, although copepods always represented the highest fraction (Fig 2B). Slight differences in the settled zooplankton composition on shells were observed between the different substrates. Bivalves, gastropods, and barnacles were all present on all shells. However, decapods were only recorded on *Anadara brasiliana*, while copepods were only recorded on *A*. *brasiliana* and *Mactra isabelleana* shells, and hydrozoan polyps were only found on *Amarilladesma mactroides* (Fig 2C). On the shells, we observed significant differences in the zooplankton colonization density (*p*<0.001) (Fig 3A). However, the richness was not affected (*p* = 0.243) (Fig 3B). No differences in the colonization on the internal and external sides of shells were observed (density *p* = 0.280; richness *p* = 0.111), although this factor may affect the invertebrate settlement density when interacting with the substrate (*p*<0.041). *A*. *brasiliana* followed by *M*. *isabelleana* showed higher densities and richness values of the zooplankton colonization on average compared to *A*. *mactroides* (Fig 3). A positive (r = 0.806) and significant (F_(1,13)_ = 24.132; *p*<0.001) correlation between zooplankton colonization density and the different external ornamentation was observed, with higher ornamentation values being more attractive.

Overall, regardless of the invertebrate’s composition, differences between the zooplankton colonization of the internal and external surfaces of *A*. *brasiliana* shells were observed. The inner surface had the highest average richness and was composed of primarily sedentary and vagile invertebrates. For all shell species, the sedentary and vagile fauna showed the highest density on the inner surfaces (Fig 3C).

### Experiment observation: Microbial biofilm colonization

**Fig 4:**
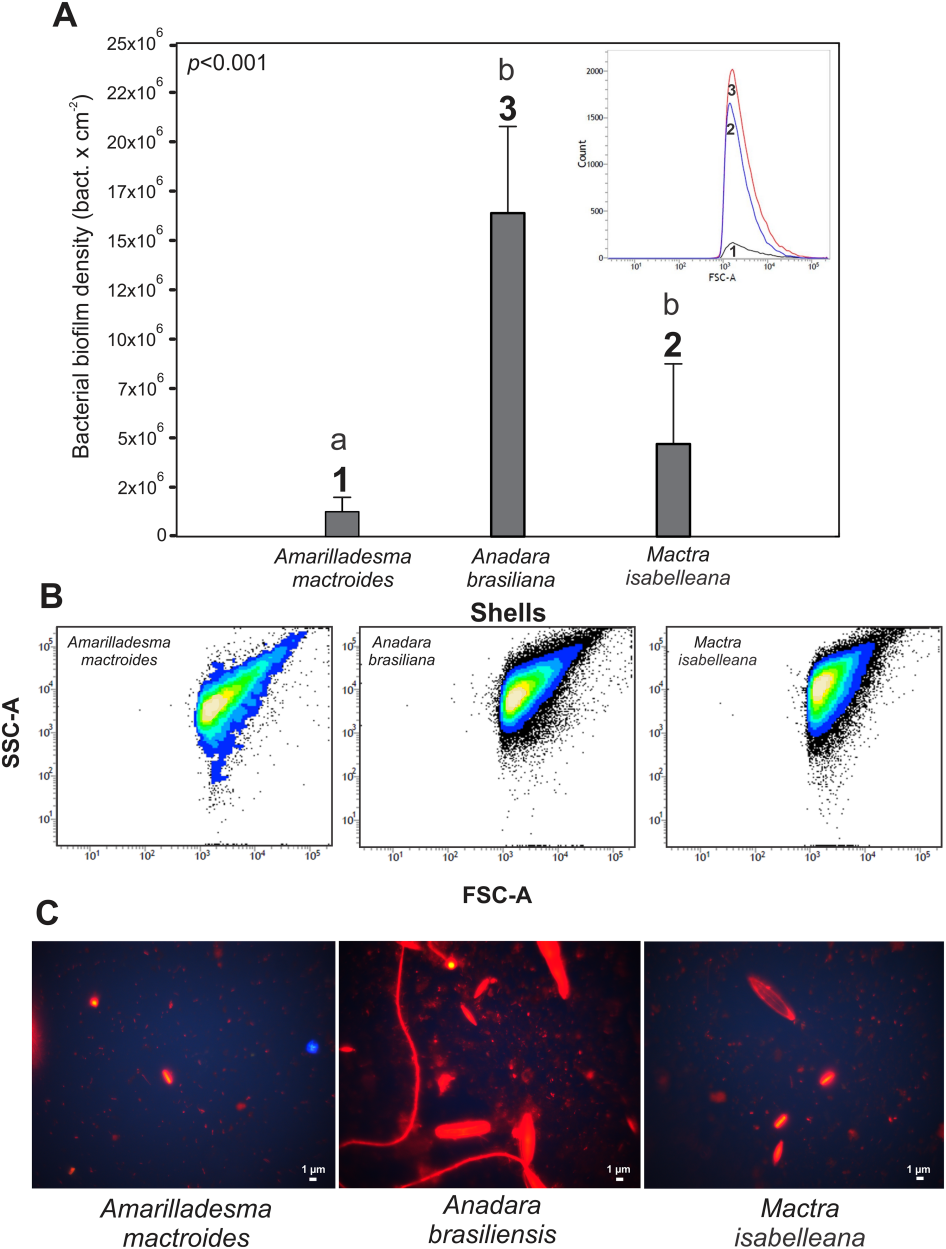
Biofilm community on shells. (A) Bacterial biofilm density (bact cm^-2^) on different shells. (B) The relative size (FSC-A) and complexity (SSC-A) of the bacterial cells measured by a flow cytometer. Each point represents a bacterial cell. The lighter colors (central part) are related to higher density cells with a determined feature (size × complexity) being characterized as one population. (C) Microorganism communities stained with acridine orange under epifluorescence microscopy (1000X). The vertical lines denote the 95% confidence intervals (standard error*1.96), and the lowercase letters indicate similarities (the same letters) or significant differences (different letters) between the shells (Tukey test).

Significant differences (*p*<0.001) were observed in the bacterial densities (bact cm^-2^) of the various bivalve species: *A*. *brasiliana* had the highest biofilm bacteria density (16.3×10^6^±2.885) followed by *M*. *isabelleana* (4.6×10^6^±32.951) and *A*. *mactroides* (1.2×10^6^ ±473.448) (Fig 4A). A positive (r = 0.896) and significant (F_(1,13)_ = 49.278; *p*<0.001) correlation between the biofilm bacteria density and the different external ornamentations of the shells was observed.

The bacterial biofilm community showed variations in cell sizes throughout the experiment (Fig 4B). *Amarilladesma mactroides* had larger bacterial cells (~0.7 μm) than the other shells. Bacteria from *A*. *brasiliana* and *M*. *isabelleana* showed an average cell size of ~0.63 and ~0.67 μm, respectively. However, the SSC-A axes from the cytometer graphs (see Fig 4B) revealed that the bacteria cells on *A*. *brasiliana* and *M*. *isabelleana* shells were more complex than the bacteria cells found on *A*. *mactroides* shells. Higher average bacterial biovolume (μm^3^) and biomass (pg C cell^-1^) values were noted on *A*. *mactroides* at 13.18 and 0.114, respectively. *Anadara brasiliana* and *M*. *isabelleana* had bacterial biovolumes of 11.87 and 12.62 μm^3^, respectively, and biomasses of 0.112 and 0.113 pg C cell^-1^, respectively. Bacterial rods and coccus shapes were observed on *A*. *mactroides* while bacterial coccus and diatoms (cf. *Nitzschia*) were observed on *M*. *isabelleana* and *A*. *brasiliana*. Filamentous fungi were also recorded on *A*. *brasiliana* (Fig 4C). A positive (r = 0.878) and significant (F_(2,27)_ = 28.352; *p*<0.001) correlation between settled zooplankton density, biofilm bacteria density and external shell ornamentation was observed (S4 Fig).

### Field observations: Mollusk assemblages

Of the 1,965 time-averaged mollusk shells (58 gastropods and 1,907 bivalves) collected from Concheiros Beach, only 828 showed sclerobionts (encrustation or bioerosion). Encrusting organisms were recorded on only 87 shells, but traces of these organisms were apparent on 741 shells. A significant difference was observed on the total sclerobiont colonization between the Bivalvia and Gastropoda classes (*p*<0.001). Table 2 presents a complete list of the bivalve and gastropod species with their relative abundances.

**Table 2:** Categorical classification of external ornamentation, mineralogy (1 = calcite; 2 = aragonite; 3 = bimineralic) and frequency of occurrence (FO) data.

Taxonomic classification	External ornamentation	Mineralogy	FO (%)
**GASTROPODA**			
*Pisania* sp.	3	2	31
*Buccinanops cochlidium*	1	2	1
*Sinum* sp.	0	2	5
*Adelomelon brasiliana*	1	2	3
*Crepidula protea*	1	2	3
*Olivancillaria urceus*	0	2	2
*Epitonium georgettinum*	3	2	2
Unidentifiable	not applicable	not applicable	32
**BIVALVIA**			
*Mactra* sp.	2	1	45.8
*Pitar* sp.	1	1	10.6
*Glycymeris* sp.	2	1	4.7
*Perna perna*	2	3	4.4
*Ostrea* sp.	2	2	1.6
*Anadara brasiliana*	3	1	1.4
*Amiantis purpurata*	2	1	0.8
*Donax* sp.	1	1	0.8
*Crassostrea* sp.	2	2	0.7
*Chlamys* sp.	3	2	0.3
*Amarilladesma mactroides*	0	1	0.1
*Brachidontes rodriguezii*	2	3	0.1
*Laevicardium* sp.	1	1	0.1
*Pholas* sp.	2	1	0.1
Unidentifiable	not applicable	not applicable	28.5

**Fig 5:**
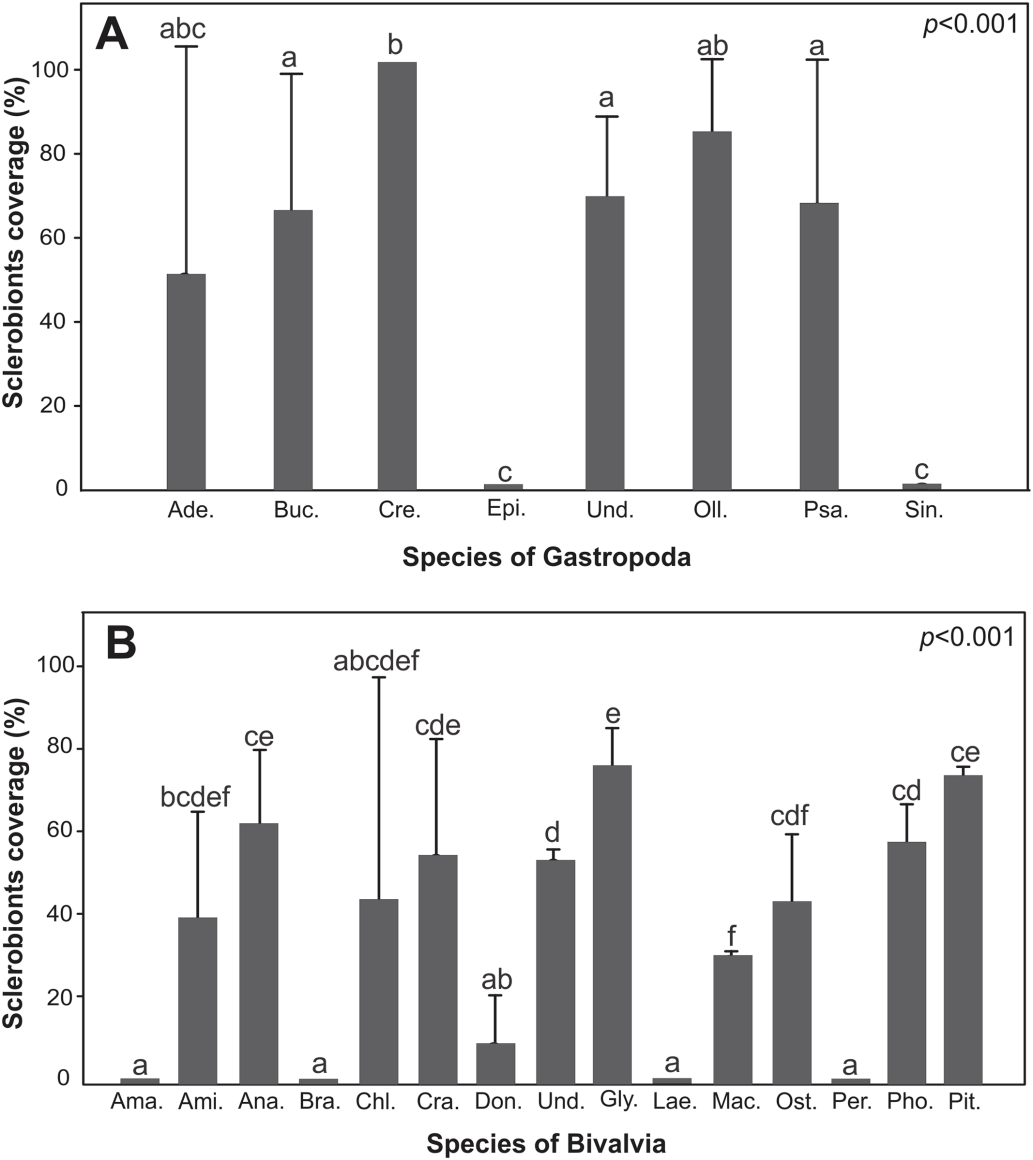
Sclerobionts coverage on mollusks. (A) Gastropoda genera: Ade.: *Adelomelon*, Buc.: *Buccinanops*, Cre.: *Crepidula*, Epi.: *Epitonium*, Oli.: *Olivancillaria*, Psa.: *Psania*, Sin.: *Sinum*. (B) Bivalvia genera: Ama.: *Amalarillodesma*, Ami.: *Amiantis*, Ana.: *Anadara*, Bra.: *Brachidontes*, Chls: *Chlamys*, Cra.: *Crassostrea*, Don.: *Donax*, Gly.: *Glycymeris*, Lae.: *Laevicardium*, Mac.: *Mactra*, Ost.: *Ostrea*, Per.: *Perna*, Pho.: *Pholas*, Pit.: *Pitar*. Und.: Unidentifiable.

The sclerobiont colonization was significantly different between the Gastropoda (*p*<0.001) (Fig 5A) and Bivalvia species (*p*<0.001) (Fig 5B). The shells of *Crepidula* spp. and *Glycymeris* spp. exhibited the highest number of sclerobionts among the Gastropoda and Bivalvia, respectively. Shells from the gastropods *Epitonium* sp. and *Sinum* sp., as well as the bivalves *Amarilladesma mactroides*, *Brachidontes* sp., *Laevicardium* sp., and *Perna perna*, showed no encrusting or bioeroding organisms.

**Fig 6:**
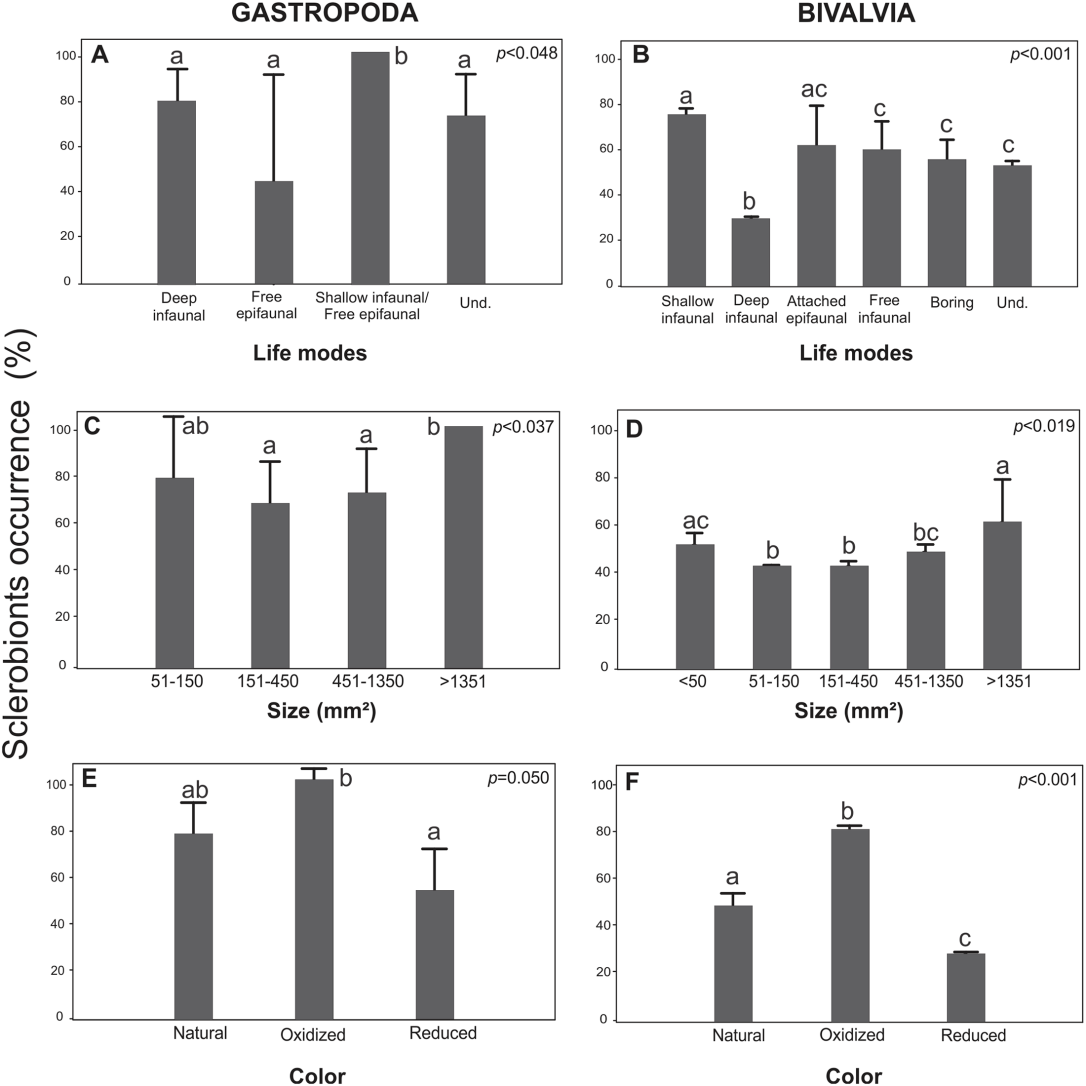
The occurrence of sclerobionts exposed to distinct life modes, sizes, and colors of the host substrates. (A) Gastropod life modes. (B) Bivalvia life modes. (C) Gastropod sizes (D) Bivalvia sizes. (E) Gastropod color. (F) Bivalvia color. Und.: Unidentifiable. The vertical lines denote the 95% confidence intervals (standard error*1.96), and the lowercase letters indicate similarities (the same letters) or significant differences (different letters) between the factors evaluated (Tukey test).

The life modes and host sizes significantly (*p*<0.048) influenced the occurrence of sclerobiont colonization (encrusters and bioeroders) on gastropods (Fig 6A and 6C) and bivalves (*p*<0.001) (Fig 6B and 6D). The shallow infaunal and attached epifaunal mollusks showed greater levels of colonization, which contrasted with the deep infaunal bivalves, which had fewer sclerobionts. Apparently, color alteration of the substrate affects sclerobiont colonization on gastropod (*p*<0.050; Fig 6E) and bivalve (*p*<0.001; Fig 6F) shells, as the oxidized (cream, yellow, ochre, or red) shells were preferentially colonized.

**Fig 7:**
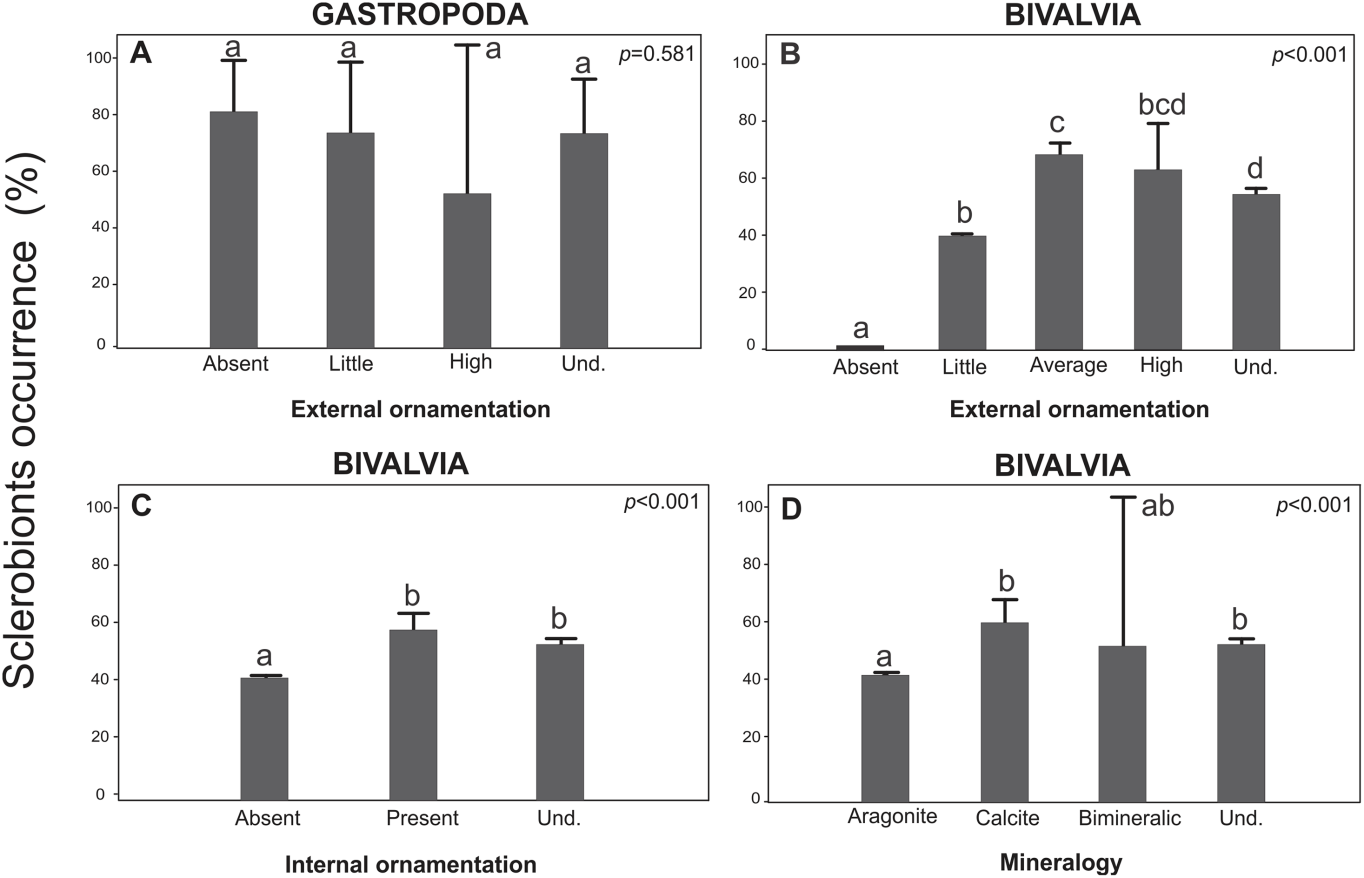
The occurrence of sclerobionts exposed to distinct ornamentation and mineralogy of the host substrates. (A) Gastropod external ornamentation. (B) Bivalvia external ornamentation. (C) Bivalvia internal ornamentation. (D) Bivalvia mineralogy. Und.: Unidentifiable. The vertical lines denote the 95% confidence intervals (standard error*1.96), and the lowercase letters indicate similarities (the same letters) or significant differences (different letters) between the factors (Tukey test).

The varying levels of external ornamentation in Gastropoda did not show any remarkable influence on sclerobiont colonization (*p* = 0.581) (Fig 7A). In contrast, the ornamentation of bivalve shells seems to be a key factor controlling the colonization process. Shells with average and high degrees of external ornamentation complexity have significantly (*p*<0.001) more sclerobionts than the bivalve shells with low degrees ornamentation complexity (Fig 7B), and the same pattern was recorded on the internal surfaces, (*p<*0.001; Fig 7C). The shell mineralogy also influenced colonization, with significantly more encrustation and bioerosion occurring on bivalve shells composed predominantly of calcite (*p*<0.001; Fig 7D).

**Fig 8:**
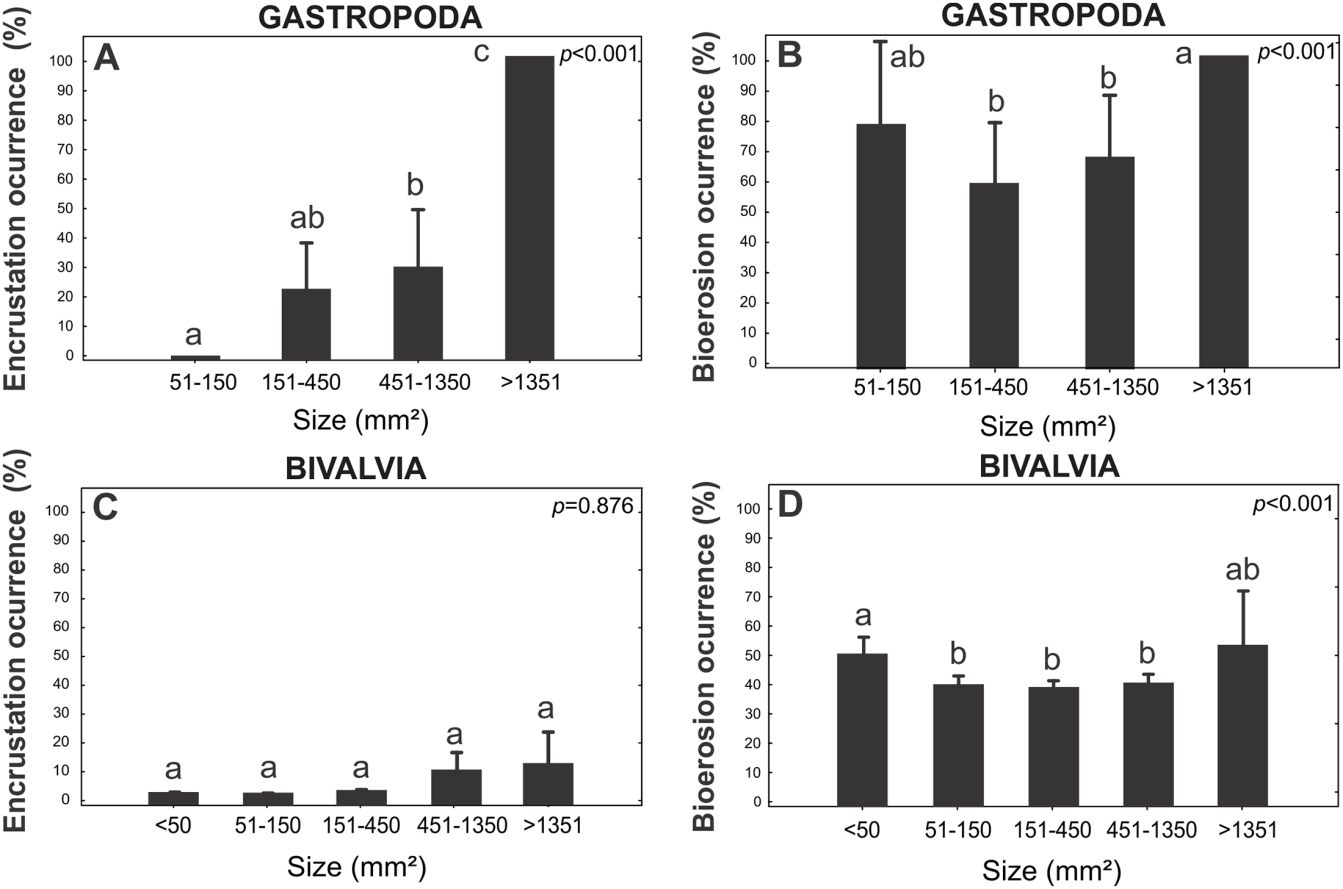
Sclerobiont occurrence on different shell size. (A) Encrustation occurrence on Gastropoda. (B) Bioerosion occurrence on Gastropoda. (C) Encrustation occurrence on Bivalvia. (D) Bioerosion occurrence on Bivalvia.

Despite these vital roles of these differences, most of the factors analyzed are covariates (Table 3). Size is a key factor, which is significantly correlated with all variables, including taphonomic damage. Size is positively correlated with color and total taphonomic grade, while it is negatively correlated with external ornamentation and mineralogy. Thus, for both gastropods and bivalves, a higher average colonization was observed on shells larger than 1,351 mm^2^ (gastropods p<0.037; bivalves p<0.019), while no significant differences were observed in the smaller size classes (51–150 mm^2^ for gastropods and <50 mm^2^ for bivalves). When bioerosion of the molluscan size classes was analyzed separately from encrustation, this pattern remained the same. However, encrustation occurred preferentially on large (>1351 mm^2^) gastropod shells (p<0.001), but no significant difference was observed for bivalve shells (p = 0.876) (Fig 8). The size frequency distributions of each taxonomic group (S3 Fig) and the taphonomic outcomes (S5, S6, S7 and S8 Figs) are displayed in the supplementary data.

**Table 3:** Spearman rank correlations between the shell factors evaluated (Table 1), and the total taphonomic grade (TTG) (see also S1 Table).

VARIABLES	Size	Color	External ornamentation	Mineralogy	TTG
**Life mode**	r = -0.460	r = -0.001	r = 0.768	r = 0.871	r = 0.162
	*p*<0.001	*p*<0.938	*p*<0.001	*p*<0.001	*p*<0.001
**Size**		r = 0.116	r = -0.407	r = -0.445	r = -0.111
		*p*<0.001	*p*<0.001	*p*<0.001	*p*<0.001
**Color**			r = 0.070	r = -0.015	r = 0.454
			*p* = 0.002	*p*<0.509	*p*<0.001
**External ornamentation**				r = 0.866	r = 0.376
				*p*<0.001	*p*<0.001

**Fig 9:**
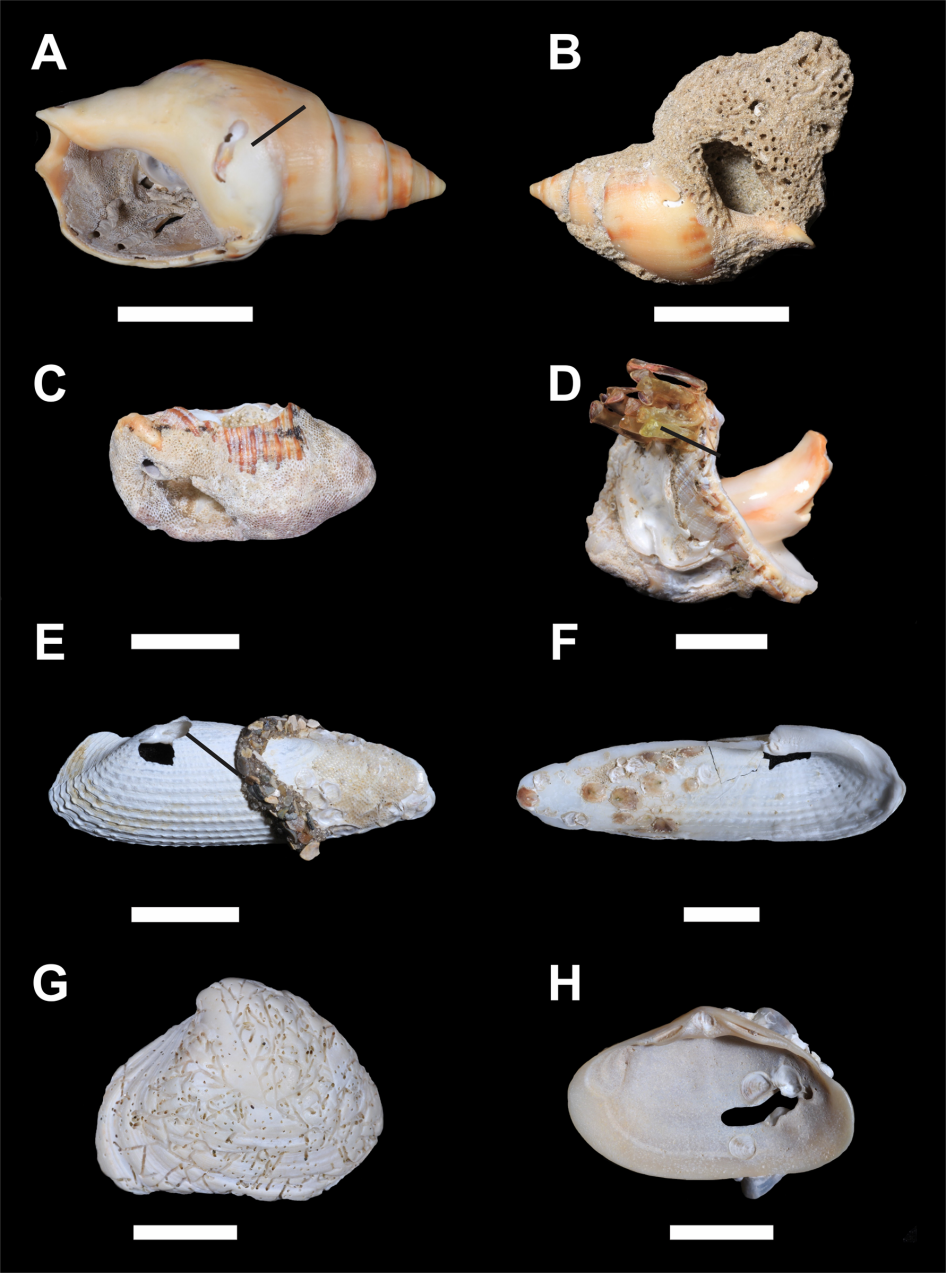
Some examples of sclerobionts that colonized molluscan shells gathered from Concheiros Beach, on the Southern coast of Rio Grande do Sul, Brazil. (A) The inside of a *Buccinanops* gastropod that contained sclerobionts, such as serpulid polychaete, bryozoans, and an oyster. On the external side of the shell, there is evidence of bioerosion by Spionidea polychaeta (arrow). (B) A gastropod shell with a sand structure made by the polychaete *Phagmatopoma caudata*. (C) A gastropod covered by bryozoans. (D) A fragment of a gastropod that was fouled by eggs of the bivalve *Stramonita haemastoma* (arrow). (E) An external view of a *Pholas* bivalve shell with encrusting Ostreidae and bryozoans and a sediment-tube made by a polychaete (arrow). (F) Internal view of another *Pholas* shell with several oysters (*Ostrea equestris*) and bryozoans. (G) External view of an *Anadara brasiliana* bivalve shell with a boring sponge. (H) Internal view of a *Mactra* bivalve shell encrusted by Ostreidae. The hole was made by a spionidea polychaete. Scale bars: 10 mm.

Several sclerobiont taxa were found colonizing the shells: *Ostrea equestris* Say, 1834; serpulid polychaetes; *Phragmatopoma caudata* Krøyer in Mörch, 1863; *Amphibalanus improvisus* (Darwin, 1854); *Stramonita haemastoma* (Linnaeus, 1767) eggs; *Crassostrea* spp.; *Pododesmus rudis* (Broderip, 1834), mytilid byssus; seaweed; Hydrozoa; Foraminifera; Bryozoa; and bioeroding Bryozoa, Porifera, Polychaeta and Bivalvia (Fig 9).

## Discussion

### Are zooplankton and biofilm bacteria colonization affected by different shells?

The zooplankton richness potential corresponded to the settled organisms on the shells, with the meroplanktonic larvae being the most representative (Fig 2A, 2B and 2C). However, the settlement quantity did not reflect the meroplankton supply. As previous studies have demonstrated, larvae are attracted to light during settlement [62, 63]. Therefore, due to the glass walls of the bowls used in our study, some larvae (i.e., barnacles) settled on the glass, thereby reducing the colonization potential on the shells.

Additionally, one meroplanktonic group that was available in the water column, the polychaetes, did not settle on the shells. This phenomenon may relate to the spatial competition with other groups that are more efficient in the settlement process [64], preference for other substrate types [46] or orientations [65], absence of conspecifics [66] or even succession of ecological needs [67], all of which can also explain the different group colonization between the bivalve shells species. For example, Marshall and Keough [68] observed that smaller larvae attach faster and are less selective than larger ones, and once one organism is present, it may influence the active choice of the substrate by others using chemical signaling.

In the experimental approach, the highest encrustation density was observed on *Anadara brasiliana* shells, which was probably due to the larger number of microhabitats available for zooplankton colonization compared to *Amarilladesma mactroides* and *Mactra isabelleana* shells. According to Carl et al. [30], surface microtopography can either induce or repel the larval settlement of many marine organisms. We observed higher invertebrate colonization on bivalve shells with texture (Fig 7). The microtopography had a strong effect in mytilids, where 400 μm (lower heterogeneity) textures enhanced the settlement at a rate of >90%. On the other hand, larger or smaller topographies led to a much-reduced colonization, which corroborated the work of Berntsson et al. [26, 27] who showed that microtopographies between 30 and 45 μm inhibited colonization by barnacle cyprids (Cirripedia) by up to 92%. This pattern might explain the absence of sclerobiont colonization on the shells of the *Epitonium* spp. gastropod, which have a high topography (ridges a few μm away from each other). Additionally, this genus is an epifaunal predator that spends time buried in the sand between feedings (i.e., partially infaunal). Intrinsic shell features, such as lower morphological heterogeneity (external ornamentation), may also account for this phenomenon.

The presence of sclerobionts on shell interiors is related to post-mortem colonization [5], which confirms the importance of this kind of substrate on sandy shelves. The encrusting taxon richness showed no differences among shells, possibly because larvae can settle on any surface type. The higher settlement densities of encrusting species recorded on *A*. *brasiliana* shells suggest that larvae have a stronger affinity to heterogeneous substrates (presence of microtopographies), and consider the external surface of the shell. In contrast, vagile/sedentary species were recorded at higher densities on shell interiors. The concave position (internal area exposed) of molluscan shells may provide protection to settling organisms since they do not attach firmly to the substrate when compared to encrusting forms.

The substrate texture can also affect the bacteria colonization [69, 70]. Thus, a similar pattern was observed regarding the shells external ornamentation when the bacterial biofilms were analyzed. A positive relationship was observed between bacteria and settled zooplankton on bivalve shells. Heterogeneous substrata (e.g., *A*. *brasiliana* shells) exhibit higher bacteria densities. It is known that surface roughness increases bacterial adhesion [71], as surface features are essential to microbiological binding to a surface [72] and bacterial attachment is independent of groove size and is greatest in the valley areas of the grooves [73]. However, in the current study, the observed bacterial densities values for the three bivalve species are within an order of magnitude, being further studies needed to corroborate this pattern. Additionally, a more complex microbial community, with diatoms and fungi were also observed on *A*. *brasiliana*, indicating a more mature biofilm, with greater bacterial biomass compared to those present on *A*. *mactroides* and *M*. *isabelleana* shells. The small differences in the bacterial sizes observed on the shells of *A*. *mactroides*, *M*. *isabelleana*, and *A*. *brasiliana* can be explained by the space competition (related to bacterial density) between bacteria cells, which allows for the various size increments of the bacteria cells [74]. Thus, the higher the bacterial density, the smaller the bacterial size.

The relationship between bacterial biofilms and the colonization of invertebrates on hard substrates (e.g., vessels, pipelines, piers) is already known [75, 76; 42]. This relationship, however, has not been proposed for settlement on shells. Contrary to the statement on biofilm production Rodland et al. [18], the formation of a polymeric matrix over the internal and external surface of a shell may attract zooplankton, and consequently enhance the colonization probabilities of sclerobioic organisms (S4 Fig). According to Tamburri et al. [77], some oyster larvae species prefer natural substrates (e.g., other oyster shells) covered with biofilms for settlement. In addition, old shells are probably less attractive to larvae for settlement rather than fresh shells, as described as the “fresh shell syndrome” by Brett et al. [7].

The shell texture influenced both the zooplankton and the bacteria colonization. However, we believe that the bacteria biofilm exerts a greater effect on the settlement of invertebrates (r = 0.828; r^2^ = 0.687) than the substrate texture (r = 0.806; r^2^ = 0.660), given the correlation values obtained.

### Is the experimental sclerobiont colonization pattern preserved on mollusk assemblages?

The taphonomic alteration on mollusk beach assemblages could be a substantial bias concerning the preservation of sclerobiont frequency [50]. However, the sclerobiont colonization in dead (as in fossil) molluscan shells appears to remain almost intact despite the taphonomic biases [9, 18]. Thus, taphonomic alteration in our data also does not play a significant role in sclerobiont colonization preservation on hosts (S5, S6, S7 and S8 Figs).

Sclerobiont colonization was more intense on shells with oxidized color, which was likely due to their taphonomic alteration (see Tables 1 and 3). Except for this study, information about the effect of the color of the substrate on bacteria and zooplankton colonization has been limited. Dobretsov et al. [78] investigated the effects of substratum color (black and white) on the formation of micro and macrofouling communities and verified that higher densities were observed on black hosts. Yule and Walker [79] and Monteforte and Garcia-Gasca [80] described the same patterns in barnacles and oysters, respectively. These findings can be explained as a result of the negative phototaxis of larvae [81], or the quantity of energy (absorbed or reflected) and the consequent temperature of the substratum [78]. These works emphasized the importance of substratum color on the formation of micro and macrofouling communities, as corroborated in this study (see Fig 6E and 6F). In the experimental approach, we only tested the colonization on white (reduced or natural colors) shells, which proved that the sclerobiont colonization could occur even in this situation. However, these results do not confirm the preference by oxidized shells from a modern perspective.

These shells have therefore revealed a complex taphonomic profile of preservation: oxidized shells were related to ancient shorelines and shallow areas [82]. Thus, most of the oxidized shells were produced by subaerial exposure during the sea level oscillations that have occurred since the Last Glacial Maximum [82]. Hence, this pattern might be related to the durability of the shells in the TAZ [1], which should increase the probabilities of larvae settlement, posterior encrustation and bioerosion. However, the relationship between color and temporal mixing has not been empirically demonstrated. Furthermore, high frequency of encrustation is not inevitably related to the colonization window time (but see Rodland et al. [18]). Thus, our results indicate that oxidized color exhibited higher frequencies of encrustation and bioerosion, or shells with color alterations were more prone to preserve the encrusters/bioeroders on their shells than those displaying reduced colors.

The mollusk assemblages are time-averaged and display the present-day ages up to ~56 kyrs on the adjacent inner shelf (but, the Holocene shells are numerically dominant; [83]). Thus, these shells have experienced different time-windows regarding the sclerobiont colonization process. Obviously, the larvae pool has not been constant or taxonomically homogenous along the time-averaging windows present in these death assemblages. Although encrustation is considered an instantaneous event (snapshot) (limited-exposure scenario *sensu* Rodland et al. [18]), older shells do not exhibit the current higher encrustation intensities or richness when compared to younger shells [18]. However, it is difficult to determine at what moment in this time-averaging window each sclerobiont settled since it is theoretically possible to find an almost infinite number of non-contemporaneous organisms. However, long-term experiments have shown that encrustation is established mainly in the first year, and the addition of new taxa decreases with time [7].

Additionally, due to the “fresh shell syndrome” [7], shells attain much of their potential coverage in the first few months; then the possibility of time-averaging of the biotic communities is probably reduced. Thus, even the settling process is a geologically instantaneous event, and the temporal acuity is limited to the host age, due to the analytical time-averaging [84]. Theoretically, any shell in a death assemblage possesses the same colonization potential when available at the seafloor, regardless of its age and taphonomic condition. Therefore, we believe that these factors will have a null effect when the encrustation on shells with a wide age range [83] is empirically tested.

The surface area plays a different role on colonization, as seen in Fig 6 and corroborates the findings in Rodland et al. [18]. We observed no differences between the shells with small (<50 mm^2^ for bivalves and 150 mm^2^ for gastropods) and large areas (greater than 1351 mm^2^) when considering encrustation and bioerosion together, or these factors separately (Fig 8). When considering encrustation, the pattern observed for gastropods was the same as that detected by Rodland et al. [5], where larger shells exhibited more severe encrustation. However, larger bivalve shells are not necessarily susceptible to greater colonization because of their larger surface areas. On the other hand, it remains unknown to what degree encrustation affects smaller or fragmented shells, as this evidence may be erased due to taphonomic processes that occur during the (wide) time-averaging window, as noted by Rodland et al. [18]. In addition, it was difficult to state that bioerosion acted directly on small shells and fragments; larger bioclasts may be bioeroded, encrusted and further fragmented, thereby losing their encrusters and only retaining their record of bioerosion. This phenomenon may explain why either smaller (fragmented shells) or larger sizes displayed the greater frequencies of sclerobiont colonization (Fig 6C and 6D). In the experiment, all shells were smaller than 50 (mm^2^), which made a comparison impossible. However, the highest invertebrate densities and richness values were found on the smallest *A*. *brasiliana* shells while the biggest shells (*A*. *mactroides*) had the lowest colonization, which was explained by their lack of external texture related to their life mode (covariables).

As shell size plays a major role in sclerobiont colonization, the significant correlation of shell size with all other factors highlights that size class is negatively correlated with taphonomic damage (Table 3). However, bigger shells showed slightly higher alterations than small shells (S5 Fig). Thus, since sclerobiont colonization is higher in bigger shells with slightly higher taphonomic bias, it confirms that taphonomic alteration does not negatively influence the preservation of sclerobiont traces on shells. Meanwhile, small fragments also displayed high intensities of sclerobionts. This finding is probably due to the fragmentation of the colonized bigger shells.

Regardless of these biases, the *Anadara* shells had the third highest occurrence of sclerobionts (%) (Fig 5A), thus, reinforcing the results of this experiment. Therefore, shell size is one of the most crucial factors [19], with external ornamentation also playing a secondary role, as experimentally demonstrated. It is difficult to account for this key element (except for shell size) since mineralogy and life modes are also correlated with external ornamentation. Remarkably, calcitic bivalves are more prone to encrustation or bioerosion. This difference may be due to the high occurrences of Ostreidae colonization by other species of the same family. Additionally, the occurrence of sclerobionts is greater in shallow infaunal species rather than epifaunal species. Some of the shallow infaunal bivalve species, such as *Glycymeris* and *Pitar*, showed a higher frequency of sclerobionts than *Amarilladesma*, a deep infaunal and relatively unornamented bivalve. Nevertheless, veneroid and myoid bivalves evolved siphons in the early Mesozoic and invaded the deep infauna [85] and are well represented in this study by the relatively ornamented genus *Pholas*. However, the shells of *Pholas* displayed an occurrence of sclerobionts comparable to *Anadara* shells, an epifaunal bivalve. Counter-intuitively, the mode of life and the mineralogy are unlikely to play key processes alone. In the experiment, we observed bacteria and zooplankton colonization on all bivalve shells, and all of these shells also show aragonite mineralogy.

Interestingly, after the Marine Mesozoic Revolution (MRV) [86], bivalves declined in the sediment column, which is well known as an infaunalization trend due to gastropod predation [87, 88]. Meanwhile, external ornamentation probably also reflects the mode of life on infaunal bivalves, which enhances its stability near the sediment-water interface [89]. External ornamentation also showed a positive correlation with a taphonomic alteration (Table 3, S5 Fig). This correlation may be an indication of a megabias in the fossil record, as relatively more ornamented species do not have higher preservability [90], but they also presented greater occurrence of sclerobionts, thus diminishing their preservability potential due to bioerosion. This finding could indicate that either shallow infaunal bivalve species are more prone to be not preserved or that sclerobiont colonization is a negatively taphonomical bias that reduces the preservability of those species. However, encrustation could be a positive bias, which increases the preservability of ornamented species. Thus, sclerobiont colonization could be a two-way bias in the fossil record needing more attention in the future.

Bivalve and gastropods shells showed differences in the factors that affected the sclerobiont occurrence. For example, a larger external texture on the gastropod shells did not proportionally reflect a greater colonization observation, nor did its mineralogy. One of the hypotheses in this study proposed a relationship between these factors and other factors (mode of life, color, taphonomic damage). These factors were hypothesized to that overlap with one another as covariates affecting the invertebrate colonization. The other hypothesis raised is related to the use of gastropods shells as housing for the vagile fauna (i.e., hermit crabs). Shell used as housing for vagile fauna are in constant movement, thereby preventing meroplankton settlement. This pattern is already observed for different substrates and is associated with hydrodynamic stress [91]. According to Walker [92], crab-inhabited shells show more encrusting organisms which could also be explained by the possible alterations caused by the hermit crabs on the gastropod shells that repel sclerobiont colonization.

## Conclusions

1. Zooplankton colonizes different shells, but the density and richness values are affected by the attributes of *Amarilladesma mactroides*, *Anadara brasiliana*, and *Mactra isabelleana* shells. Additionally, fouling invertebrates seem to be more associated with the external shell sides, while vagile and sedentary fauna are more associated with the internal side.
2. The external shell texture seems to directly affect the bacteria biofilm density, as most ornate surfaces are more attractive. Zooplankton colonization seems to respond directly to bacteria density, the microbial biofilm community, and consequently to the external ornamentation of the shells.
3. Shell size is one of the most significant variables regarding sclerobiont colonization, as previous studies have documented. External ornamentation also plays at least a secondary role, as experimentally demonstrated. However, all factors may have a covarying effect on sclerobiont occurrence on the shells.
4. The sclerobiont occurrence patterns observed for bivalves do not apply in the same way to gastropods (external ornamentation and life mode), which is probably related to other factors that were not evaluated.
5. Similar sclerobiont patterns were also found in experimental and assemblage deposit observations, despite the taphonomic biases. These observations allowed us to infer that an experiment might be used to explain the paleontological patterns. However, as our study has covered only three bivalve species experimentally, broader studies are still necessary.

## Supporting information

S1 Data Raw data on zooplankton abundance used in the analyses in this study. (XLSX)

S2 Data Biofilm density data used in this paper. (XLSX)

S3 Data Taphonomic scores of all shells from “Concheiros” Beach, Southern Brazil. The table presents the raw data of the taphonomic scores of 1,965 shells (58 gastropods and 1,907 bivalve shells) used in this paper. See also S4 Data and S1 Table.
(CSV)

S4 Data A more detailed description of the methods used (taphonomic analyses). (DOCX)

S1 Table Taphonomic protocol utilized in this study. (DOCX)

S1 Fig Species employed in the study screening for different external textures. (A) *Amarilladesma mactroides* (Reeve 1854), external view. (B) *Amarilladesma mactroides*, internal view. (C) *Mactra isabelleana* d'Orbigny 1846, external view. (D) *Mactra isabelleana*, internal view. (E) *Anadara brasiliana* (Lamarck 1819), external view. (F) *Anadara brasiliana*, internal view. Scale bars: 5 cm.
(TIF)

S2 Fig Experimental diagrams employed in both the laboratory and the experimental field steps of the current study. (A) Zooplankton colonization experiment. Each bowl (20 cm in diameter, 18 cm in height) was filled with estuarine water up to a height of 10 cm and kept at a constant salinity (23±2), temperature (25°C), and photoperiod (14L:10D). These conditions were preferred to simulate the subtropical conditions found in this region. A 5 cm-thick layer of natural sediment was included as substrate at the bottom of each bowl to simulate the upper limit of the taphonomically active zone. (B) The field experiment in the channel of the Patos Lagoon estuary in Southern Brazilian.
(TIF)

S3 Fig Size-frequency distributions for each mollusk class collected. (A) Gastropoda. (B) Bivalvia.
(TIF)

S4 Fig Multiple regression analysis between bacterial density (bact cm^-2^) and zooplankton colonization density (org 25 cm^-2^) regarding the external ornamentation of shells. (TIF)

S5 Fig Total taphonomic grade (percentage damage index) of intrinsic measured variables in Bivalvia. **The box plots are showing interquartile range, the 95% confidence intervals and the outliers**. (A) Size class. (B) External ornamentation. (C) Mineralogy. (D). Life mode. All *p*-values were obtained from the Kruskal-Wallis Test. Und.: undetermined.
(TIF)

S6 Fig Total taphonomic grade (percentage damage index) of the intrinsically measured variables in Gastropoda. **The box plots are showing the interquartile range, the 95% confidence intervals and the outliers**. (A) Size class. (B) Life mode. (C) External ornamentation. All p-values were obtained from the Kruskal-Wallis Test. Und.: undetermined.
(TIF)

S7 Fig Total taphonomic grade (percentage damage index) among Bivalvia species. **The box plots are showing the interquartile range, the 95% confidence intervals and the outliers**. Bivalvia genera: Ama.: *Amalarillodesma*, Ami.: *Amiantis*, Ana.: *Anadara*, Bra.: *Brachidontes*, Chls: *Chlamys*, Cra.: *Crassostrea*, Don.: *Donax*, Gly.: *Glycymeris*, Lae.: *Laevicardium*, Mac.: *Mactra*, Ost.: *Ostrea*, Per.: *Perna*, Pho.: *Pholas*, Pit.: Pitar. Und.: Unidentifiable. *p*-value was obtained from the Kruskal-Wallis Test.
(TIF)

S8 Fig Total taphonomic grade (percentage damage index) among Gastropod species. **The box plots are showing the interquartile range, the 95% confidence intervals and the outliers**. Gastropoda genera: Ade.: *Adelomelon*, Buc.: *Buccinanops*, Cre.: *Crepidula*, Epi.: *Epitonium*, Oli.: *Olivancillaria*, Psa.: *Psania*, Sin.: *Sinum*. (B). *p*-value was obtained from the Kruskal-Wallis Test.
(TIF)
