# Supplementary material for: What determines sclerobiont colonization on marine mollusk shells?
Source: PLoS One. 2017 Sep 13;12(9):e0184745. doi: 10.1371/journal.pone.0184745 (PMC5597280; doi:10.1371/journal.pone.0184745)
Supplement: S4 Data — (DOCX) [file pone.0184745.s004.docx]

**SD Data 4**

**What determines sclerobiont colonization on marine mollusk shells?**

Vanessa Ochi Agostini, Matias do Nascimento Ritter, Alexandre José Macedo, Erik Muxagata and Fernando Erthal

**Taphonomic analyses**

All shells observed herein (n= 1965) were assigned damage states using a categorical scoring system established and modified from previous workers (Best 2008; Ritter et al. 2013 and references therein) (Table SI 1, SI Data 3). For taphonomic analysis, specimens were observed under low (10-20 X) magnification using a stereoscopic microscope, and binned for variables describing fragmentation, fine-scale surface alteration, and color alteration (Table SI 1). Many terms for fine-scale surface alteration have been applied, but ones used here are descriptive and do not allude to a one only process (Best 2008 and references therein). In our protocol, consequently, fine-scale alteration denotes various degrees of degradation of original luster; it may be due to any combination of microboring and other microbioerosion, partial dissolution of mineral crystallites, maceration of shell organic matrix, and physical abrasion, which need a scanning electron microscope (SEM) to distinguish (Best 2008).

Preceding analysis, the taphonomic profile (scoring damage for each signature on each shell) was standardized, dividing the row sum by the maximum value (equal to numbers of taphonomic attributes counted). Thus, the taphonomic profile for each shell will be ranged between 0 (pristine) and 100 percentage of damage (maximum taphonomic alteration). In order to test for differences in the standardized taphonomic damage among variables, we performed the Kruskal-Wallis Test.
