# Supplementary material for: What determines sclerobiont colonization on marine mollusk shells?
Source: PLoS One. 2017 Sep 13;12(9):e0184745. doi: 10.1371/journal.pone.0184745 (PMC5597280; doi:10.1371/journal.pone.0184745)
Supplement: S1 Table — (DOCX) [file pone.0184745.s005.docx]

**SI Table 1** — Taphonomic protocol utilized in this study.

| **Taphonomic attribute** | **Scores** | **More information** |
| --- | --- | --- |
| Fragmentation  Fine-scale surface (FSA) alteration  Secondary color* (or color alteration) | 0 = absent  1 = present  0 = pristine  1 = present  1.1 = small pits 1.2 = large pits  1.3 = small and large pits  1.4 = holes 1.5 = small pits and holes  1.6 = large pits and holes  1.7 = small and large pits and holes  0 = color lost; 1=natural; 2 = oxidized color; 3 = reduced color. | Zuschin et al. (2003)  Best (2008) and Ritter et al. (2013)  Best (2008) |
| *oxidized color (cream, yellow, ochre, and red); reduce color (white, gray and black) | | |
