# Supplementary figures and images for: What determines sclerobiont colonization on marine mollusk shells?
Source: PLoS One. 2017 Sep 13;12(9):e0184745. doi: 10.1371/journal.pone.0184745 (PMC5597280; doi:10.1371/journal.pone.0184745)

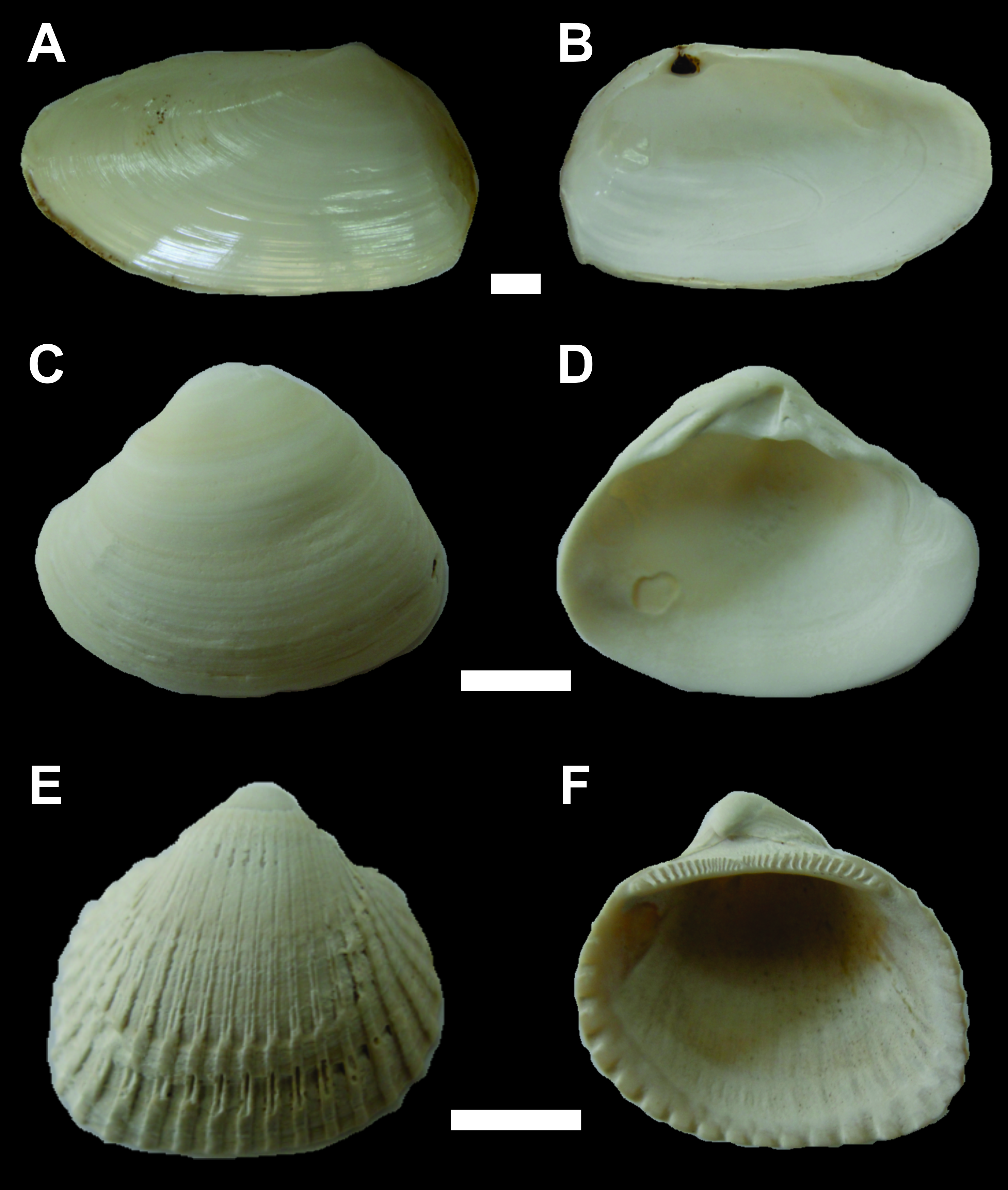

Supplement: S1 Fig — (A) Amarilladesma mactroides (Reeve 1854), external view. (B) Amarilladesma mactroides, internal view. (C) Mactra isabelleana d'Orbigny 1846, external view. (D) Mactra isabelleana, internal view. (E) Anadara brasiliana (Lamarck 1819), external view. (F) Anadara brasiliana, internal view. Scale bars: 5 cm. (TIF) [file pone.0184745.s006.tif]

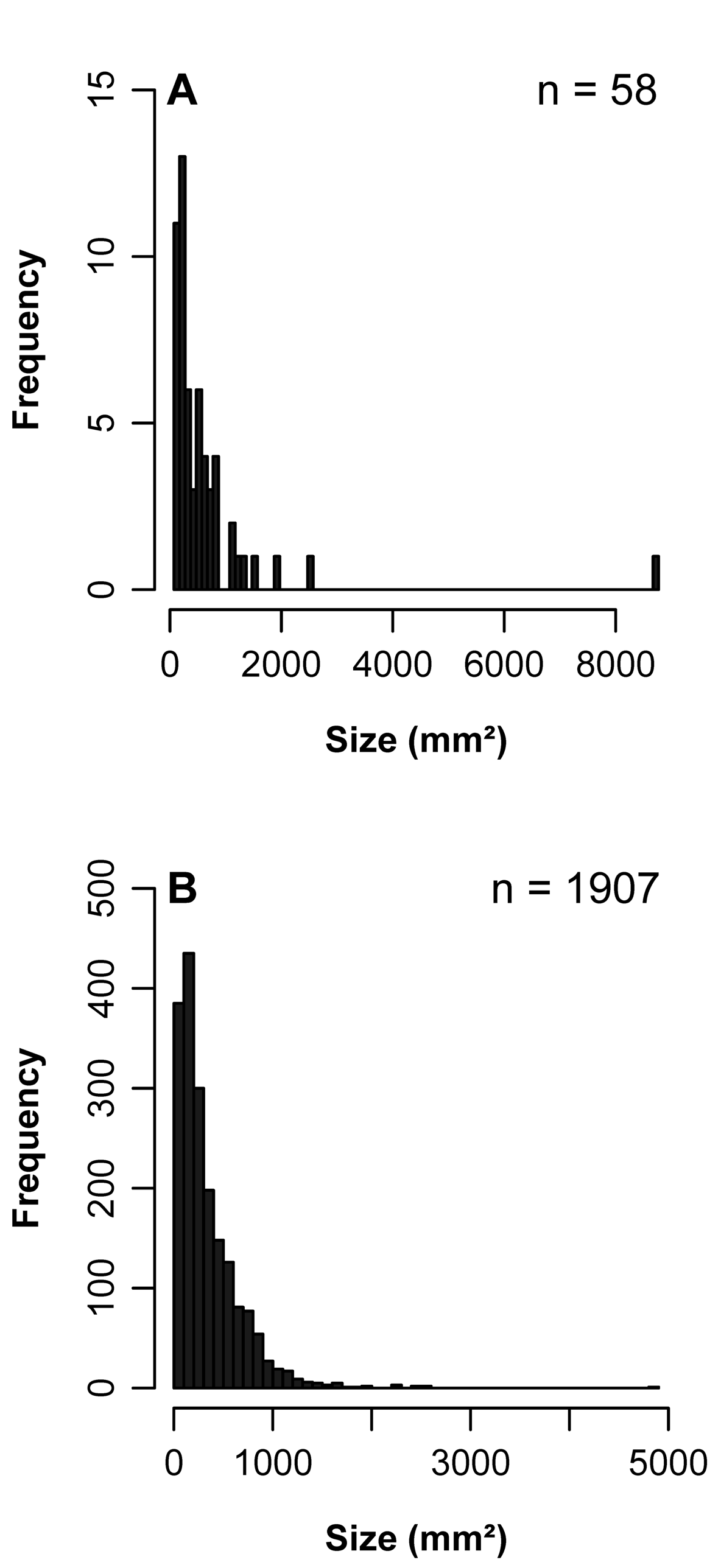

Supplement: S3 Fig — (A) Gastropoda. (B) Bivalvia. (TIF) [file pone.0184745.s008.tif]

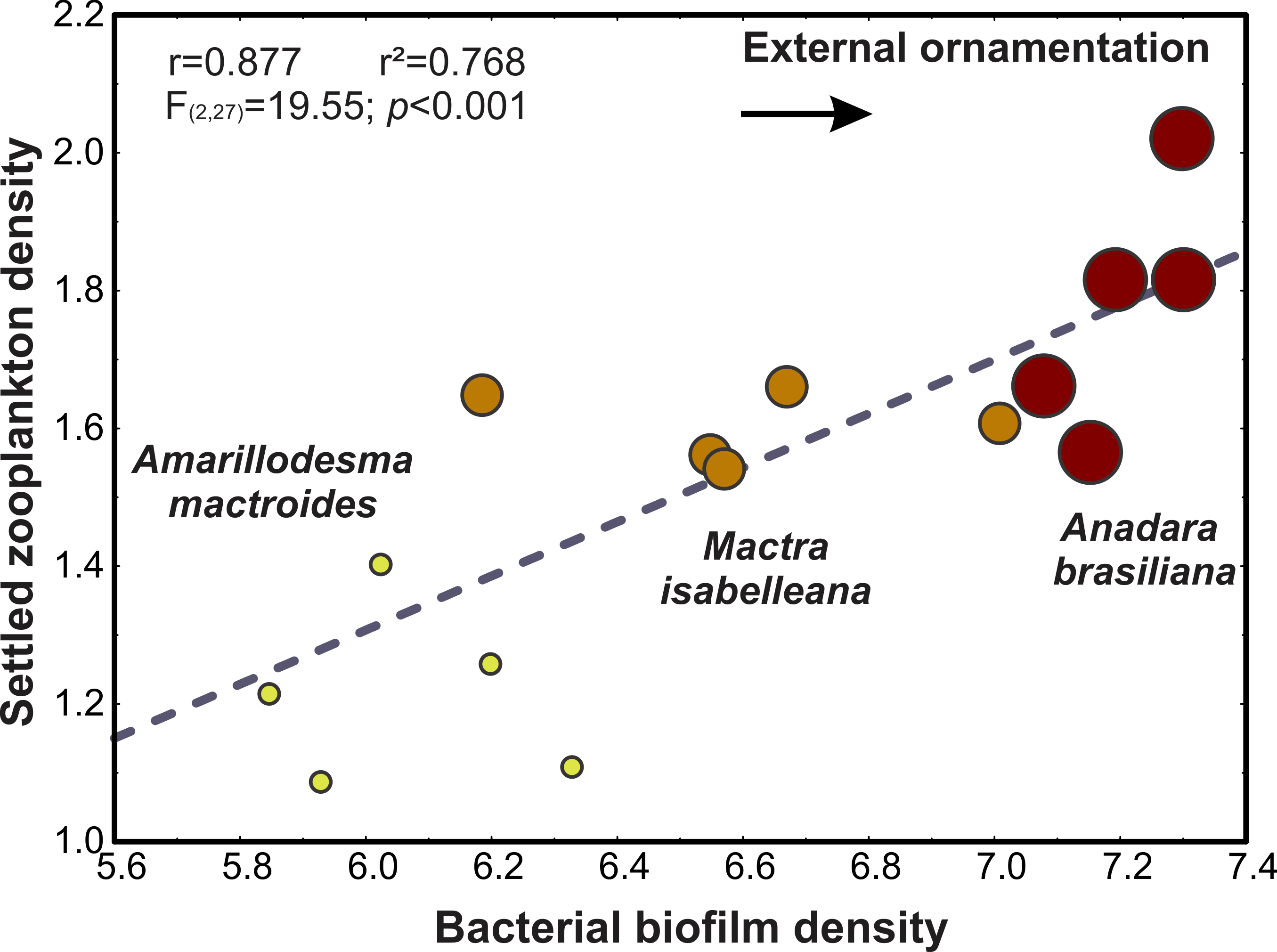

Supplement: S4 Fig — (TIF) [file pone.0184745.s009.tif]
